# Up-regulating the abscisic acid inactivation gene *ZmABA8ox1b* contributes to seed germination heterosis by promoting cell expansion

**DOI:** 10.1093/jxb/erw131

**Published:** 2016-03-31

**Authors:** Yangyang Li, Cheng Wang, Xinye Liu, Jian Song, Hongjian Li, Zhipeng Sui, Ming Zhang, Shuang Fang, Jinfang Chu, Mingming Xin, Chaojie Xie, Yirong Zhang, Qixin Sun, Zhongfu Ni

**Affiliations:** ^1^State Key Laboratory for Agrobiotechnology and Key Laboratory of Crop Heterosis and Utilization (MOE), Beijing Key Laboratory of Crop Genetic Improvement, China Agricultural University, Beijing 100193, China; ^2^National Plant Gene Research Center (Beijing), Beijing 100193, China; ^3^National Center for Plant Gene Research (Beijing), Institute of Genetics and Developmental Biology, Chinese Academy of Sciences, Beijing 100101, China; ^4^National Maize Improvement Center of China, China Agricultural University, Beijing 100193, China

**Keywords:** Abscisic acid, gene expression, heterosis, maize, seed germination, *ZmABA8ox1b*.

## Abstract

Heterosis has been widely used in agriculture, but the underlying molecular principles are still largely unknown. During seed germination, we observed that maize (*Zea mays*) hybrid B73/Mo17 was less sensitive than its parental inbred lines to exogenous abscisic acid (ABA), and endogenous ABA content in hybrid embryos decreased more rapidly than in the parental inbred lines. *ZmABA8ox1b*, an ABA inactivation gene, was consistently more highly up-regulated in hybrid B73/Mo17 than in its parental inbred lines at early stages of seed germination. Moreover, ectopic expression of *ZmABA8ox1b* obviously promoted seed germination in *Arabidopsis*. Remarkably, microscopic observation revealed that cell expansion played a major role in the ABA-mediated maize seed germination heterosis, which could be attributed to the altered expression of cell wall-related genes.

## Highlight

*ZmABA8ox1b*-mediated abscisic acid inactivation increases seed germination rate by promoting cell expansion in the maize hybrid B73/Mo17 compared with its parental inbred lines.

## Introduction

Heterosis, or hybrid vigour, refers to the superior performance of hybrids relative to the parental lines (Shull, 1914). Although heterosis is extensively used in breeding programmes to increase crop production, the underlying molecular mechanism is poorly understood. It has been documented that plant growth and development are often determined by global gene expression networks (Long *et al.*, 2008). Although all of the genes in the F_1_ hybrid are derived from its parental inbred lines, hybrid performance is often markedly different from that of either parent. This is probably because the altered gene expression in hybrids contributes to heterosis (Sun *et al.*, 2004; Chen, 2010). Recently, several differentially expressed genes (DEGs) between hybrids and their parental inbred lines have been reported to play an important role in plant growth and development, such as *ZmEBP1* (Wang *et al.*, 2016), *ZmACT2* (Guo *et al.*, 2013), *LaAP2L1* (Li *et al.*, 2013), and *ZmCNR1* (Guo *et al.*, 2010). However, the function of DEGs in heterosis requires further investigation.

Seed germination represents the developmental transition from maturation drying to a sustained metabolic rate in preparation for seedling establishment. Germination is also considered to be a critical and sophisticated process in the plant life cycle that is strictly controlled by endogenous and environmental signals (Nonogaki *et al.*, 2010; Weitbrecht *et al.*, 2011; Rajjou *et al.*, 2012). It is well known that F_1_ hybrid seeds have a superior germination capacity compared with their parental inbred lines, but the underlying molecular mechanism remains unclear (Schnable and Springer, 2013). At the gene expression level, studies have indicated that the vigorous growth of the embryonic axis in germinating F_1_ seeds is related to a higher rate of RNA and protein synthesis (Romagnoli *et al.*, 1990). Ding *et al.* (2012) reported that the global repression of microRNAs in the maize (*Zea mays*) hybrid Yuyu22 might result in enhanced gene expression and thus explain the higher embryo germination vigour compared with its parental inbred lines.

Abscisic acid (ABA) regulates many physiological processes, such as seed maturation, seed dormancy and germination, and adaptive responses to environmental stresses (Zeevaart and Creelman, 1988; Hoffmann-Benning and Kende, 1992; Nambara and Marion-Poll, 2005). ABA levels are controlled by two key regulatory steps: carotenoid cleavage by 9-cis-epoxycarotenoid dioxygenase (NCED) and ABA inactivation by ABA 8′-hydroxylase (CYP707A) (Nambara and Marion-Poll, 2005; Seo *et al.*, 2009; Arc *et al.*, 2013). Moreover, genetic mutants of ABA-responsive transcription factors, such as *ABA INSENSITIVE 3* (*ABI3*), *ABI4*, and *ABI5*, exhibited reduced ABA sensitivity and accelerated seed germination in the presence of exogenous ABA (Finkelstein and Somerville, 1990). Notably, proteomic analysis of different hybrid combinations during maize seed germination showed that ABA and gibberellin regulation networks were involved in seed germination heterosis (Fu *et al.*, 2011). Considering the importance of ABA in seed germination, the alteration of ABA metabolic and signalling pathways may regulate seed germination heterosis, particularly during radicle emergence, which deserves further study.

Maize is one of the most widely utilized model systems for heterosis-based studies, including the elucidation of genetic mechanisms underlying heterosis (Flint-Garcia *et al.*, 2009). Maize F_1_ hybrid plants are taller, display increased biomass, grow more rapidly, and have greater yields than their inbred parents (Flint-Garcia *et al.*, 2009). Notably, one of the key aspects of maize heterosis is accelerated seed germination (Fu *et al.*, 2011). Although microRNAome, transcriptome, and proteome analyses have contributed greatly to our understanding of seed germination heterosis in maize, the regulation of seed germination and its heterosis is incompletely explored (Fu *et al.*, 2011; Ding *et al.*, 2012; Guo *et al.*, 2013). Here, we identified and characterized the hybrid up-regulated ABA inactivation gene *ZmABA8ox1b*. The expression level of this gene was highly correlated with rapid ABA inactivation, which led to a phenotype of lower sensitivity to exogenous ABA in hybrid B73/Mo17 compared with its parental inbred lines during seed germination. *Arabidopsis* transformation analysis further confirmed that *ZmABA8ox1b* participated in regulating ABA inactivation and seed germination. Moreover, microscopic observation revealed that cell expansion was responsible for the ABA-mediated maize seed germination heterosis, and this process may be attributed to the altered expression of cell wall-related genes in hybrid plants revealed by RNA-seq analysis.

## Materials and methods

### Plant materials and seed germination

One highly heterotic hybrid, B73/Mo17, its female parent, B73, and its male parent, Mo17, were selected for this study. Germination efficiency was determined in triplicate. For each replicate, 50 uniform seeds were placed embryo-side down in Petri dishes (90mm in diameter) containing two layers of filter paper and 12ml of distilled water. Plates were then placed in a 28°C growth chamber in the dark. Seeds were considered to be germinated when radicle protrusion was visible. Seed germination was scored regularly for 20–68h. ABA (Sigma-Aldrich, USA) and oryzalin (Sigma-Aldrich) were dissolved in DMSO (Sigma-Aldrich) as stock solutions and diluted to the appropriate working concentrations in water when needed. Controls contained the same volume of DMSO.

The time to 50% germination (*T*_50_) was used to evaluate the rate of seed germination and was calculated using a previously established formula (Coolbear *et al.*, 1984): *T*_50_ = *t*_*i*_ + (*t*_*j*_ − *t*_*i*_) × (*N*/2 − *n*_*i*_)/(*n*_*j*_ − *n*_*i*_). This index uses the weighted mean between hours *t*_*i*_ and *t*_*j*_ and the cumulative seed counts (*n*_*i*_ and *n*_*j*_) adjacent to half of the total sum of germinated seeds (N/2) so that *n*_*i*_ < N/2 < *n*_*j*_.

### RNA extraction and quantitative reverse transcription PCR

The germination of a batch of maize seeds was not strictly synchronous; therefore, at least 15 imbibed embryos of each genotype were dissected and mixed for RNA extraction. Total RNA was extracted using a polysaccharide and polyphenol plant total RNA isolation kit (BioTeke, China) according to the manufacturer’s instructions. In addition, rosette leaves of 3-week-old 35S::*ZmABA8ox1b Arabidopsis* lines and wild type (Col-0) were collected and total RNA was extracted using a standard Trizol RNA isolation protocol (Invitrogen, USA).

Quantitative reverse transcription (qRT)-PCR was performed as previously described (Guo *et al.*, 2013). Briefly, ~2 μg of total RNA from each sample was reverse transcribed to cDNA using Reverse Transcription Reagent (TaKaRa, Japan) with an attached Oligo(dT)15 primer according to the manufacturer’s instructions. qRT-PCR was performed using a CFX96 Real-Time PCR Detection System (Bio-Rad Laboratories, Inc., USA) with a SYBR Green PCR master mix (TaKaRa). *ZmActin1* (GRMZM2G126010) and *AtActin2* (AT3G18780) were amplified as endogenous controls for maize and *Arabidopsis*, respectively. The specific primers used to detect transcripts are listed in Supplementary Table S1.

### Quantification of endogenous ABA

Embryos were isolated from the seeds of hybrid B73/Mo17 and its parental inbred lines 0, 4, 8, 12, and 16 hours after imbibition (HAI). Seeds of *Arabidopsis* 35S::*ZmABA8ox1b* lines and wild type were harvested and stored at room temperature for 2 weeks. The amount of ABA was measured using the above embryos and dry seeds, with three biological replicates. For each replicate, at least 15 frozen maize embryos or 50mg dry *Arabidopsis* seeds were homogenized in liquid nitrogen, and 200mg of the homogenized fresh weight of maize and 10mg of *Arabidopsis* seeds were used to measure ABA. The samples were then extracted for 24h with cold methanol (−20°C) containing 0.3mM antioxidant and 6ng ^2^H_6_-ABA (internal standard; OlChemIm Ltd, Czech Republic). Endogenous ABA was purified and measured as previously described (Fu *et al.*, 2012) with changes in detection conditions. The ultra-performance (UP) LC–MS/MS system consisted of an UPLC system (ACQUITY UPLC^TM^, Waters, USA) and a hybrid triple quadrupole–linear ion trap mass spectrometer (QTRAP 5500; AB SCIEX, USA). The chromatographic separation was achieved on a BEH C_18_ column (50mm × 2.1mm, 1.7 μm; Waters) with a column temperature of 25°C and a flow rate of 0.2ml/min.. The linear gradient ran from 95% to 85% A (solvent A, 0.05% acetic acid aqueous; solvent B, acetonitrile) for 1min, 85% to 30% A for the next 5min, and 30% to 2% A for the following 1min, before re-equilibration with the initial condition for 2min. The optimized MS parameters included a curtain gas at 40 psi, collision gas at 6 psi, an ion spray voltage of −4300V, and temperature of 550°C. The declustering potential was −85V and the collision energy was −15V. The multiple reaction monitoring mode was used for quantification, and the selected multiple reaction monitoring transitions were 263.0→153.1 for ABA and 269.1→159.3 for ^2^H_6_-ABA.

### Vector construction and plant transformation

The 35S::*ZmABA8ox1b* and pOp6::*ZmABA8ox1b* constructs were created using the Gateway cloning system (Invitrogen) by cloning the full-length *ZmABA8ox1b* coding sequence into the pDONR221 cloning vector and then into the binary vectors pG2BW7 (Invitrogen) and pKIGW, separately. The specific primers used for PCR amplification are listed in Supplementary Table S1. All constructs were verified by DNA sequencing. The resulting plasmids were introduced into the *Agrobacterium tumefaciens* strain GV3101. The plasmids were subsequently transformed into *Arabidopsis* wild type according to the floral dip method (Clough and Bent, 1998). Transgenic plants were screened using herbicides (Basta, Bayer CropScience, Tokyo, Japan) or kanamycin-containing medium. Homozygous lines carrying a single insertion in the T3 generation were used for further analysis.

### Histological analysis

The embryo radicles of hybrid B73/Mo17 and its parental inbred lines were dissected at 16 and 24 HAI with or without 200 μM ABA treatment. These embryo radicles were fixed in formalin-acetic acid-alcohol mixtures and sent to Guge Biotechnology Co., Ltd (Beijing, China) for embedding, slicing, and toluidine blue staining. These sections were examined using a Nikon Inverted Microscope. The cell length of two or three layers of intact cortical parenchyma cells was measured with three biological replicates each, and each replicate contained at least three embryo radicles.

### RNA deep sequencing and data analysis

The embryos at 16 HAI were isolated from the seeds of hybrid B73/Mo17 and its parental inbred lines for RNA deep sequencing; three replicates were performed. RNA-seq libraries were prepared using the Illumina TruSeq RNA Library Preparation Kit v2 (Illumina, USA) according to the manufacturer’s protocol, and ~36 GB 100-bp paired-end reads were generated on an Illumina HiSeq platform. The FastQC program (http://www.bioinformatics.babraham.ac.uk/projects/fastqc/) was initially run to assess the overall quality of the RNA-seq reads. Poor-quality bases were filtered out using Sickle (Joshi and Fass, 2011). RNA-seq reads were aligned to the maize B73 reference genome AGPv3 (Schnable *et al.*, 2009) using Splice Junction Mapper TopHat2 version 2.0.9 (Kim *et al.*, 2013). HTseq (http://www-huber.embl.de/users/anders/HTSeq/doc/overview.html) was used to determine the read counts mapped to each gene. The read counts were normalized to reads per kilobase of transcript per million mapped reads (RPKM) (Mortazavi *et al.*, 2008) to determine the relative level of expression. The bioconductor package edgeR (Robinson *et al.*, 2010) was used for differential expression analysis. The genes showing an absolute value of log2 (fold change) ≥1 and an false discovery rate-adjusted *P*-value <0.05 were considered to be differentially expressed. The expression patterns were defined using established criteria: (i) ‘above high inbred parent expression’ is when expression in the hybrid is significantly higher than that in both parental inbred lines; (ii) ‘high inbred parent expression’ is when expression in the hybrid is equal to that in the high expression parent but significantly different from that in the low expression parent; (iii) ‘low inbred parent expression’ is when expression in the hybrid is equal to that in the low expression parent but significantly lower than that in the high expression parent; (iv) ‘below low inbred parent expression’ is when expression in the hybrid is significantly lower than in both parents; (v) ‘partial dominance’ is when expression in the hybrid is significantly higher than that in the low expression parent and significantly lower than that in the high expression parent; (vi) ‘additive expression’ is when expression in the hybrid is equal to the mid-parent value (the average of the two parental inbred lines); and (vii) ‘different’ is an expression pattern that does not fit in any of the above expression patterns.

### Statistical tests

Student’s *t*-tests were conducted using the Excel software. The least significant difference (LSD) test and two-way ANOVA were conducted using the SPSS software (version 21.0 for Apple Macintosh Mac OS X). The significance thresholds are indicated in the figure legends.

## Results

### Effects of ABA on the seed germination of the maize hybrid and its parental inbred lines

**Fig. 1.**
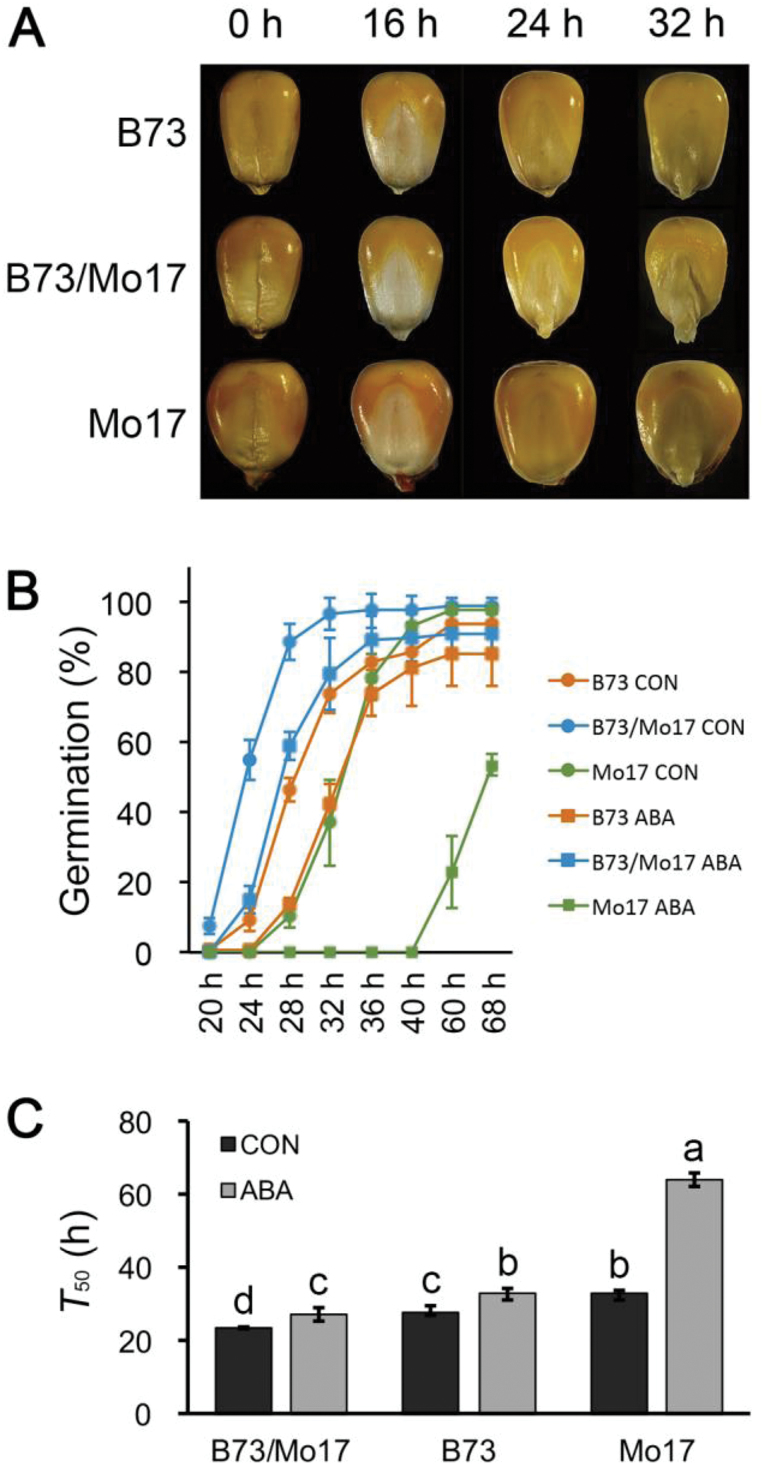
**Seed germination of hybrid B73/Mo17 and its parental inbred lines.** (**A**) Germination of hybrid B73/Mo17 and its parental inbred lines in distilled water. (**B**) Germination time course of hybrid B73/Mo17 and its parental inbred lines and the effect of 200 μM exogenous ABA on this process. ABA was not added to the control (CON). (**C**) Comparison of *T*_50_ (hours to germination of 50% of all germinated seeds) between the hybrid B73/Mo17 and its parental inbred lines with or without 200 μM ABA. Data are shown as the mean ± SD for three replicates. Each replicate included at least 50 seeds. Different letters are used to indicate means that differ significantly (*P* < 0.05, LSD test).

To investigate the relationship between ABA and maize seed germination heterosis, we focused on radicle emergence and analysed the effects of exogenous ABA on the hybrid B73/Mo17 and its parental inbred lines. As shown in Fig. 1A, radicle protrusion initiated earlier in B73/Mo17 than in its parental inbred lines in distilled water. A time-course analysis of seed germination showed that approximately 55% of hybrid seeds showed visible radicle emergence at 24 HAI compared with only 10% and 0% for B73 and Mo17, respectively (Fig. 1B). This superiority of radicle emergence in the hybrid B73/Mo17 was maintained for 40h until almost all of the seeds had fully germinated (Fig. 1B). When exogenous ABA (200 μM) was applied, the seed germination rates of the hybrid and its parental inbred lines were significantly lower than those of the respective controls (Fig. 1B). We calculated the *T*_50_ for each genotype with or without ABA treatment. After treatment with 200 μM ABA, the *T*_50_ of hybrid B73/Mo17 was 27h, the *T*_50_ of B73 was 27h, and the *T*_50_ of Mo17 was 64h, which were significantly delayed compared with their corresponding controls (24h, 28h and 33h, respectively; *P* < 0.05). Statistical analysis indicated that the *T*_50_ of parental inbred lines B73 and Mo17 was greatly increased by ABA treatment compared with that of the hybrid B73/Mo17, suggesting an interaction between ABA treatment and genotypes (Fig. 1C, two-way ANOVA, F = 353, *P* < 0.0001). This provided circumstantial evidence that the maize hybrid B73/Mo17 is less sensitive to the exogenous application of ABA.

**Fig. 2.**
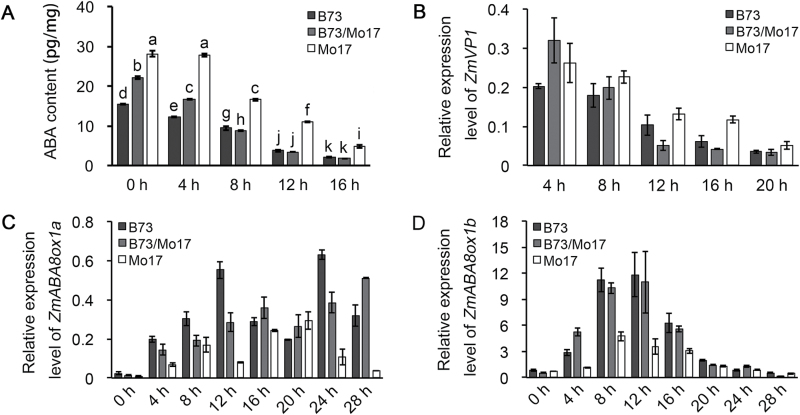
**Endogenous ABA content and gene expression level in embryos of hybrid B73/Mo17 and its parental inbred lines during seed germination.** (**A**) Endogenous ABA contents. Different letters are used to indicate means that differ significantly (*P* < 0.05, LSD test). (**B–D**) Relative expression levels of *ZmVP1* (B), *ZmABA8ox1a* (C), and *ZmABA8ox1b* (D) during seed germination. *ZmActin1* was used as an internal control. Data are shown as the mean ± SD (n = 3).

To further investigate the role of ABA in seed germination heterosis, we determined the endogenous ABA content in seed embryos of the hybrid B73/Mo17 and its parental inbred lines at 0, 4, 8, 12, and 16 HAI. Endogenous ABA content drastically decreased during seed germination (Fig. 2A). ABA content in hybrid B73/Mo17 decreased by approximately 5.36 pg/mg from 0 to 4 HAI, which was a much greater decrease than that in B73 and Mo17 (3.23 and 0.36 pg/mg, respectively) (Fig. 2A). This indicates that the decrease of ABA content in the hybrid was faster compared with its parental inbred lines at the early stage of seed germination. Moreover, ABA content in hybrid B73/Mo17 was below or equal to that in the low inbred parent at 8, 12, and 16 HAI (Fig. 2A), which may contribute to the observed heterosis in terms of radicle emergence. To explore whether the changes of endogenous ABA content influenced the ABA signalling pathway in hybrid B73/Mo17 and its parental inbred lines, we examined the expression level of *ZmVP1*, a key ABA-responsive gene in seed embryos. Consistently, the expression level of *ZmVP1* decreased during seed germination and exhibited below low inbred parent or low inbred parent expression patterns in seed embryos at 12 and 16 HAI (Fig. 2B). Collectively, we propose that this alteration of ABA inactivation plays an important role in seed germination heterosis in maize.

### Identification of the hybrid up-regulated gene *ZmABA8ox1b* and its role in ABA inactivation during seed germination

Previous studies have shown that germination in imbibed seeds is preceded by a decline in ABA that results from inactivation by ABA 8′-hydroxylase (ABA8ox) (Kushiro *et al.*, 2004; Saito *et al.*, 2004). Thus, we speculated that the difference in ABA inactivation rates between the maize hybrid and its parental inbred lines may be attributed to the altered expression of *ABA8ox*. In maize, a genome-wide analysis revealed five putative *ZmABA8ox* genes, including *ZmABA8ox1a*, *ZmABA8ox1b*, *ZmABA8ox2*, *ZmABA8ox3a*, and *ZmABA8ox3b* (Vallabhaneni and Wurtzel, 2010). We analysed the expression levels of these five *ZmABA8ox* genes in hybrid B73/Mo17 using qRT-PCR (Supplementary Table S1). Only *ZmABA8ox1a* and *ZmABA8ox1b* were expressed at detectable levels in the embryos during seed germination (Supplementary Figure S1). Next, we investigated the expression patterns of *ZmABA8ox1a* and *ZmABA8ox1b* in hybrid B73/Mo17 and its parental inbred lines (Fig. 2C, D). The expression level of *ZmABA8ox1a* was not correlated with endogenous ABA content, whereas the expression level of *ZmABA8ox1b* rapidly increased at the early stage of seed germination and then declined after 12h imbibition in both hybrid B73/Mo17 and its parental inbred lines (Fig. 2D), consistent with the rapid decrease of endogenous ABA content (Fig. 2A). Notably, *ZmABA8ox1b* displayed an above high inbred parent or a high inbred parent expression pattern at 4, 8, 12, and 16 HAI (Fig. 2D). Thus, *ZmABA8ox1b* was selected for further analysis.

**Fig. 3.**
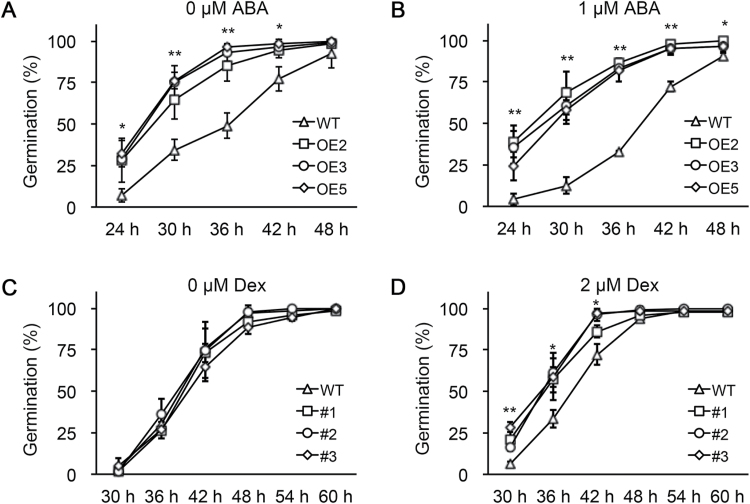
**Time course of seed germination for wild type and *ZmABA8ox1b* transgenic lines.** (**A, B**) Time course of seed germination of wild type (WT) and three 35S::*ZmABA8ox1b* transgenic lines without (A) or with (B) 1 μM ABA treatment. OE2, OE3, and OE5 represent the three 35S::*ZmABA8ox1b* transgenic lines. (**C, D**) Time course of seed germination for WT and three pOp6::*ZmABA8ox1b* transgenic lines without (C) or with (D) 2 μM Dex treatment. #1, #2, and #3 represent the three pOp6::*ABA8ox1b* transgenic lines. Data are shown as the mean ± SD for three replicates. Each replicate included at least 50 seeds. Data were statistically analysed with a *t*-test: *P < 0.05, **P < 0.01.

To investigate the function of *ZmABA8ox1b* on seed germination, a 35S::*ZmABA8ox1b* overexpression (OE) construct was introduced into wild-type *Arabidopsis*. The qRT-PCR results showed different expression levels of *ZmABA8ox1b*, but transcription of the homologous gene of *ZmABA8ox1b* in *Arabidopsis* (*AtCYP707A2*) was repressed in the 35S::*ZmABA8ox1b* OE lines (Supplementary Figure S2A, B). Next, we carried out a seed germination time-course experiment to determine the difference between transgenic lines (OE2, OE3, and OE5) and the wild type. As shown in Fig. 3A, B, the three 35S::*ZmABA8ox1b* transgenic lines displayed a markedly increased seed germination rate under 0 μM and 1 μM ABA conditions compared with the wild type. Consistent with these results, the ABA content in dry seeds of 35S::*ZmABA8ox1b* transgenic lines was significantly lower than that in wild type (Supplementary Figure S2C). To further investigate the role of *ZmABA8ox1b* in seed germination, we used the inducible pOp6/LhGR expression system. In the absence of dexamethasone (Dex), the *Arabidopsis* seed germination rate in the *ZmABA8ox1b* transgenic lines was comparable to that in the wild type (Fig. 3C). However, the seed germination rate of the pOp6::*ZmABA8ox1b* transgenic line was accelerated in Dex-containing Murashige and Skoog medium compared with the wild type (Fig. 3D). Taken together, these data reveal that the *ZmABA8ox1b* is involved in ABA inactivation and the control of seed germination in *Arabidopsis*.

### Evidence for the important role of cell expansion in ABA-regulated seed germination heterosis

**Fig. 4.**
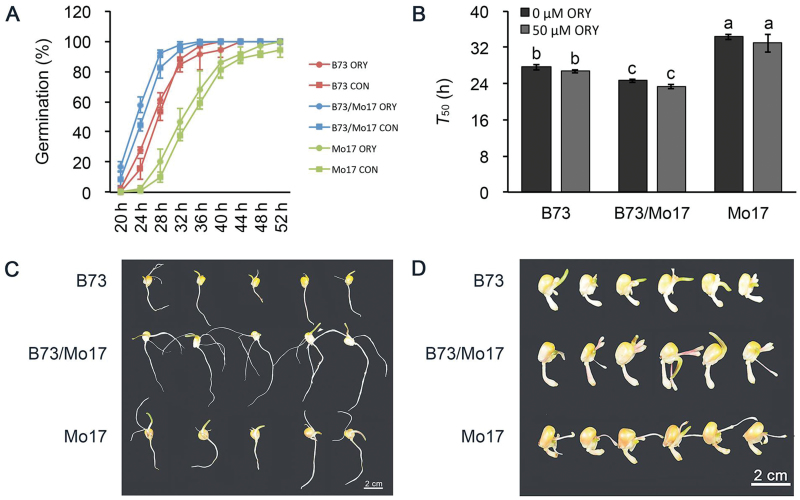
**Effects of oryzalin on seed germination in hybrid B73/Mo17 and its parental inbred lines.** (**A, B**) The time course of seed germination (A) and *T*_50_ (B) between the hybrid B73/Mo17 and its parental inbred lines with (ORY) or without (CON) 50 μM oryzalin. Data are shown as the mean ± SD for three replicates. Each replicate included at least 50 seeds. Data were statistically analysed with a *t*-test. (**C, D**) Post-germination seeding growth of the hybrid B73/Mo17 and its parental inbred lines without (C) or with (D) 50 μM oryzalin treatment at 3 days after imbibition.

Cell proliferation and expansion are two developmental forces for organ growth at the cellular level (Mizukami, 2001). Previous studies have reported that ABA regulates germination through the control of radicle emergence by inhibiting cell-wall loosening and cell expansion (Bewley, 1997). To elucidate the role of cell expansion in seed germination heterosis and its relationship to ABA, we inhibited cell division in the hybrid B73/Mo17 and its parental inbred lines during seed germination using oryzalin (Fig. 4A). The rate of seed germination (*T*_50_) for any genotype was not affected by the application of 50 μM oryzalin when compared with the control (0 μM oryzalin) (Fig. 4B). However, the continued growth of the germinated seeds was severely inhibited by oryzalin, indicating that oryzalin effectively inhibited cell division (Fig. 4C, D). Based on these results, we propose that cell expansion plays an important role in maize seed germination heterosis.

**Fig. 5.**
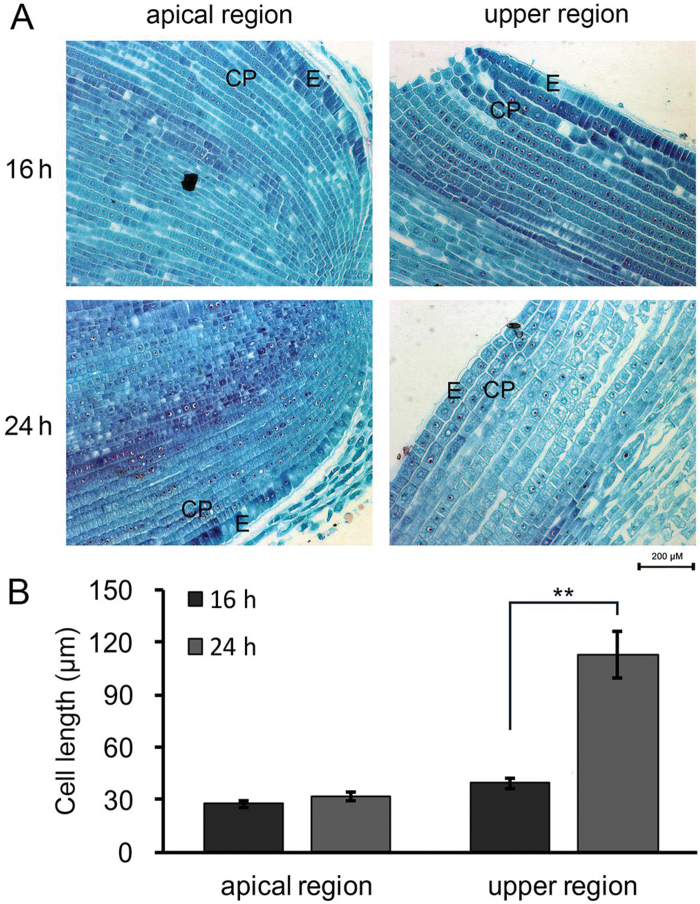
**Comparison of cell length in the apical and upper regions of the hybrid embryo radicle at 16 and 24 HAI.** (**A**) Microscopic observation of a longitudinal section of the apical and upper regions of the hybrid B73/Mo17 at 16 and 24 HAI. E, epidermis. CP, cortical parenchyma. (**B**) Measurement of cortical parenchyma cells in the apical and upper regions of the hybrid B73/Mo17 at 16 and 24 HAI. Data were statistically analysed with a *t*-test: *P < 0.05, **P < 0.01.

**Fig. 6.**
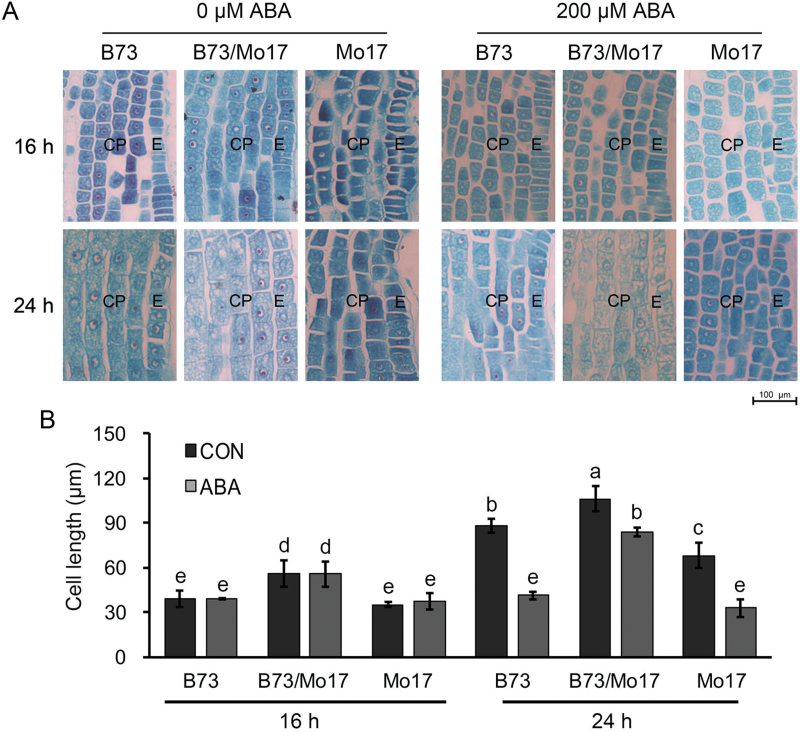
**Effect of ABA on cortical parenchyma cell expansion in the upper regions of the embryo radicle of hybrid B73/Mo17 and its parental inbred lines at 16 and 24 HAI.** (**A**) Microscopic observation of cortical parenchyma cells from the embryo radicle in the upper region of the hybrid B73/Mo17 and its parental inbred lines at 16 and 24 HAI with or without 200 μM ABA treatment. E, epidermis. CP, cortical parenchyma. (**B**) Measurement of cortical parenchyma cells in the upper regions of the hybrid B73/Mo17 and its parental inbred lines at 16 and 24 HAI with or without ABA treatment. Different letters are used to indicate means that differ significantly (*P* < 0.05, LSD test).

Subsequently, we measured and compared the cell length of the hybrid B73/Mo17 and its parental inbred lines at 16 and 24 HAI using conventional microscopy with meta-chromatic toluidine-blue colouration. Consistent with previous studies (Gimeno-Gilles *et al.*, 2009), the radial and axial growth of cells depended on the position of the embryo radicle during seed germination (Supplementary Figure S3). The cortical parenchyma cell lengths in the upper (under the hypocotyl) and apical regions of the hybrid embryo radicle had increased by 186% and 15% at 24 HAI compared with 16 HAI, respectively (Fig. 5A, B). Thus, the cell length in the upper region of the embryo radicle of the hybrid B73/Mo17 and its parental inbred lines was selected for further comparison (Fig. 6A). As shown in Fig. 6B, the cell length of hybrid B73/Mo17 at 24 HAI was much greater than that of its parental inbred lines. The effect of exogenous ABA on cell expansion was also investigated (Fig. 6A). The results showed that the cell length in the upper region of the embryo radicle of each genotype decreased after treatment with 200 μM ABA (Fig. 6B). Statistical analysis indicated that the decrease in cell length in the parents after ABA treatment was significantly larger than in the hybrid, suggesting an interaction between ABA treatment and genotypes (Fig. 6B, two-way ANOVA, F = 6, *P* < 0.05). This is consistent with the observation that the seed germination rate of hybrid B73/Mo17 was less sensitive to ABA treatment than its parental inbred lines (Figs 1C and 6B). Combined with the data regarding endogenous ABA content, we conclude that the decreased ABA content in the hybrid seed embryos leads to rapid cell expansion and influences seed germination heterosis.

### Comparative transcriptome analysis of embryos between the hybrid and its parental lines at 16h after seed imbibition

To obtain additional information about the candidate ABA-regulated genes involved in seed germination heterosis, we performed Illumina high-throughput sequencing to compare the expression profiles of the hybrid B73/Mo17 and its parental inbred lines. Based on the time lag between the changes in ABA content and the response of gene expression, seed embryos of each genotype were collected at 16 HAI for RNA-seq. Our three biological replicates were in close agreement with one another, as shown by a 0.96–0.99 Pearson correlation coefficient (Supplementary Figure S4). To further validate the accuracy of the data, we randomly selected 20 genes for qRT-PCR analysis, and 17 genes exhibited similar expression patterns to those shown by the RNA-seq data (Supplementary Figure S5). Using the edgeR program (Robinson *et al.*, 2010), we identified 5468 DEGs, accounting for ~27.28% (5468/20 042) of the expressed genes. Among these 5468 DEGs, 2792 genes showed additive expression patterns and 2676 genes exhibited non-additive expression patterns (Table 1). The DEGs from the non-additive expression pattern were classified into six categories: above high inbred parent expression (400 genes); high inbred parent expression (466 genes); low inbred parent expression (661 genes); below low inbred parent expression (278 genes); partial dominance expression (671 genes); and an expression pattern different from the mid-parent value but not belonging to any of the other classes (200 genes) (Table 1).

**Table 1.** Comparison of differential gene expression during germination between the maize hybrid B73/Mo17 and its parental inbred lines

**Hybrid cross**	**Differentially expressed pattern**								
	**Additive**	**Non-additive**							**Total**
		**＋＋** ^**a**^	**＋** ^**b**^	**＋/－** ^**c**^	D^**d**^	**－** ^**e**^	**－－** ^**f**^	**Sum**	
B73/Mo17	2792	400	466	671	200	661	278	2676	5468

^a^ ＋＋: above high inbred parent expression; ^b^ ＋: high inbred parent expression; ^c^ ＋/－: partial dominance expression; ^d^ D: different from additivity (mid-parent value), not belonging to any of the other classes; ^e^ －: low inbred parent expression; ^f^ －－: below low inbred parent expression.

**Fig. 7.**
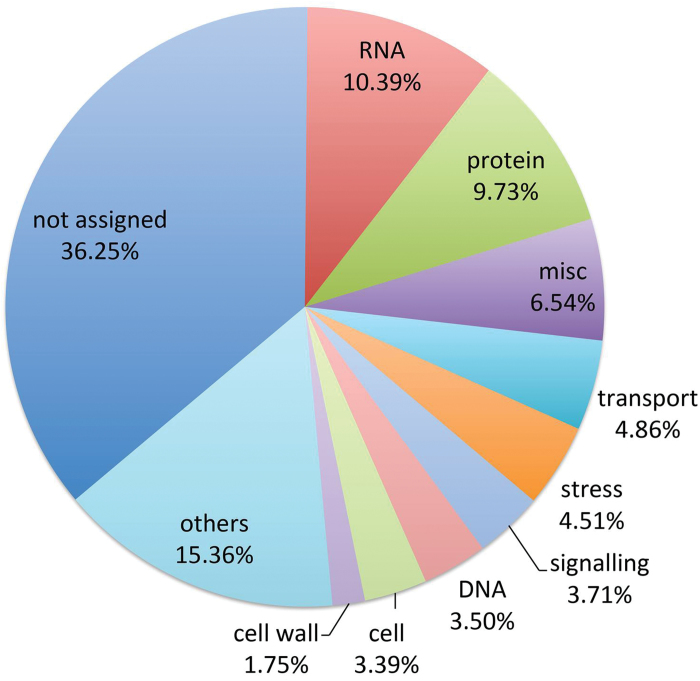
**Classification of the 2676 non-additively expressed genes according to the MapMan BINs.** The 2676 non-additively expressed genes were classified in 11 categories as indicated. The ‘others’ category includes hormone metabolism (2.66%), development (2.55%), lipid metabolism (1.85%), secondary metabolism (1.64%), amino acid metabolism (1.15%), minor CHO metabolism (0.80%), redox (0.63%), photosynthesis (0.59%), major CHO metabolism (0.49%), metal handling (0.49%), nucleotide metabolism (0.42%), N-metabolism (0.28%), co-factor and vitamin metabolism (0.28%), glycolysis (0.24%), biodegradation of xenobiotics (0.24%), tricarboxylic acid/org transformation (0.17%), tetrapyrrole synthesis (0.17%), fermentation (0.14%), mitochondrial electron transport/ATP synthesis (0.14%), oxidative pentose phosphate (0.10%), S-assimilation (0.10%), polyamine metabolism (0.10%), and C1-metabolism (0.07%).

Based on the MapMan ontology (Thimm *et al.*, 2004), the 2676 non-additively expressed genes were grouped into 11 categories. A relatively large group of genes (36.25%) could not be classified by MapMan and were labelled as ‘not assigned’. The remaining genes were divided into 35 MapMan BINs. The most abundant BIN was RNA (10.39%), the second was protein (9.73%), and the third was miscellaneous (miscellaneous enzyme families; 6.54%). The remaining BINs were transport (4.86%), stress (4.51%), signalling (3.71%), DNA (3.50%), cell (3.39%), cell wall (1.75%), and others (15.36%) (Fig. 7).

Plant cells are encased by cell walls composed of cellulose, hemicelluloses, and pectin, compounds that aid in resisting turgor pressure (Cosgrove, 2005). To prevent a progressive thinning of the wall during cell expansion, wall materials must be synthesized and added to the growing wall (Cosgrove, 2005; Somerville, 2006). Considering the important role of cell expansion in seed germination heterosis, we focused on the non-additively expressed genes related to cell wall BINs. Interestingly, 50 of the 2676 non-additively expressed genes were involved in cell wall metabolism, including precursor synthesis, cellulose synthesis, hemicellulose synthesis, cell wall proteins, modification, pectin esterases, and degradation (Supplementary Table S2). To further investigate the relationship between ABA and these non-additively expressed genes related to cell wall metabolism during seed germination, we analysed the expression levels of 18 genes with above high inbred parent or high inbred parent expression patterns before and after treatment with 50 μM exogenous ABA. The results showed that the mRNA abundance of 17 genes was decreased at 16 HAI in seed embryos after ABA treatment in both hybrid and parental inbred lines. Of these 17 genes, 13 had above high inbred parent or high inbred parent expression pattern at 16 HAI without ABA treatment. Of these 13 genes, 12 exhibited higher mid-parent heterosis (MPH) in terms of relative expression level after ABA treatment than that expressed before ABA application. Next, we analysed the expression patterns of four candidate genes during seed germination, and found that these four genes were up-regulated during seed germination and displayed above high inbred parent or high inbred parent expression patterns at 12 and 16 HAI (Supplementary Figure S6), which was consistent with the alteration of ABA content. Taken together, we propose that ABA-mediated seed germination heterosis is related to the activation of genes encoding cell wall biosynthesis and architecture.

## Discussion

### *ZmABA8ox1b*-regulated ABA inactivation contributes to seed germination heterosis in maize

The biological basis of heterosis has been of primary interest for more than a century owing to its scientific and practical importance (Schnable and Springer, 2013). Over the last decade, genetic analyses and genome-wide gene expression profiling studies have greatly advanced our understanding of the molecular mechanisms of heterosis (Stupar *et al.*, 2008; Zhang *et al.*, 2008; He *et al.*, 2010; Chodavarapu *et al.*, 2012; Shen *et al.*, 2012; Zhai *et al.*, 2013). By contrast, there is little information on the regulation of heterosis at the physiological level. In plants, all physiological aspects are affected to some extent by hormones, including auxin, gibberellin, cytokinin, ABA, and ethylene. Growing evidence has revealed that plant hormones have an important role in heterosis. For example, the enhancement of several key salicylic acid biosynthesis genes increased biotrophic pathogen resistance in *Arabidopsis* hybrids (Yang *et al.*, 2015), and gibberellin content and metabolism are positively correlated with the shoot and stem growth rate of hybrids (Zhang *et al.*, 2007; Ma *et al.*, 2011). As an essential plant hormone, ABA functions in many plant developmental processes, but its role in heterosis remains enigmatic. Here, we have demonstrated that ABA plays an important regulatory role in seed germination heterosis, which is supported by three lines of evidence: (i) endogenous ABA content in embryos of hybrids declined more rapidly than in the parental inbred lines at early stages of seed germination (Fig. 2A); (ii) the *T*_50_ of parental inbred lines B73 and Mo17 was greatly increased by ABA treatment compared with their hybrid B73/Mo17 (Fig. 1C), indicating that seed germination of parental inbred lines was more sensitive than that of the hybrid to exogenous ABA; and (iii) the ABA-responsive gene *ZmVP1* exhibited low inbred parent or below low inbred parent expression patterns in hybrid seed embryos at 12 and 16 HAI (Fig. 2B).

The ABA metabolic pathway has been extensively studied, and many genes encoding enzymes in each step of this pathway have been identified in model plant species (Nambara and Marion-Poll, 2005). Thus, investigation of the expression patterns of ABA metabolism-related genes in hybrids and their parental inbred lines will provide additional knowledge on the underlying mechanisms regulated by ABA. In *Arabidopsis*, ABA 8′-hydroxylase CYP707A2-regulated ABA inactivation plays a central role in the transition from embryonic development to germination growth (Koornneef *et al.*, 2002; Cutler *et al.*, 2010). In this study, we found that two of five ABA 8′-hydroxylase genes (*ZmABA8ox1a* and *ZmABA8ox1b*) were expressed in embryos during seed germination (Supplementary Figure S1), and the mRNA abundance of *ZmABA8ox1b* was coordinated with endogenous ABA content (Fig. 2A, D). Moreover, *ZmABA8ox1b* displayed an above high inbred parent or high inbred parent expression pattern in embryos of the hybrid B73/Mo17 at an early stage of seed imbibition (Fig. 2D). Considering the time lag between gene expression and ABA inactivation, the expression level of *ZmABA8ox1b* was positively correlated with endogenous ABA content in the hybrid and its parental inbred lines. Moreover, the ectopic expression of *ZmABA8ox1b* promoted seed germination in *Arabidopsis* (Fig. 3). We propose that *ZmABA8ox1b*-regulated ABA inactivation contributes to seed germination heterosis in maize. Notably, despite the lower expression level of *ZmABA8ox1b* in Mo17 compared with hybrid B73/Mo17 and B73, the ABA content in Mo17 also declined rapidly at later stages of seed germination. Thus, the underlying mechanism will be an interesting area for further investigation.

### ABA-mediated seed germination heterosis may be attributed to the activation of genes encoding cell wall biosynthesis and architecture

Enlarged organ size is a critical feature of heterosis (Swanson-Wagner *et al.*, 2006; Guo *et al.*, 2010; Guo and Simmons, 2011). At the cellular level, plant growth is regulated by the integration of two processes: cell proliferation and cell expansion. Previous studies have demonstrated that the larger organ size of hybrids is primarily due to an increase in cell number (Pavlikova and Rood, 1987). However, knowledge regarding the role of cell expansion in the heterosis of many specific traits is limited, especially for seed germination. In the present study, the phenotypic analysis of a maize hybrid and its parental inbred lines during germination under mitotic inhibition conditions showed that cell division was not required for radicle emergence (Fig. 4A, B). Consistent with these data, microscopic observations demonstrated that the length of cortical parenchyma cells in the upper region of the hybrid embryo axes increased faster than those in its parental inbred lines at early stages of seed germination (Fig. 6B). Collectively, these data reveal that cell expansion plays a central role in seed germination heterosis, which is in contrast to findings from previous studies on organ size heterosis (Guo *et al.*, 2010).

**Fig. 8.**
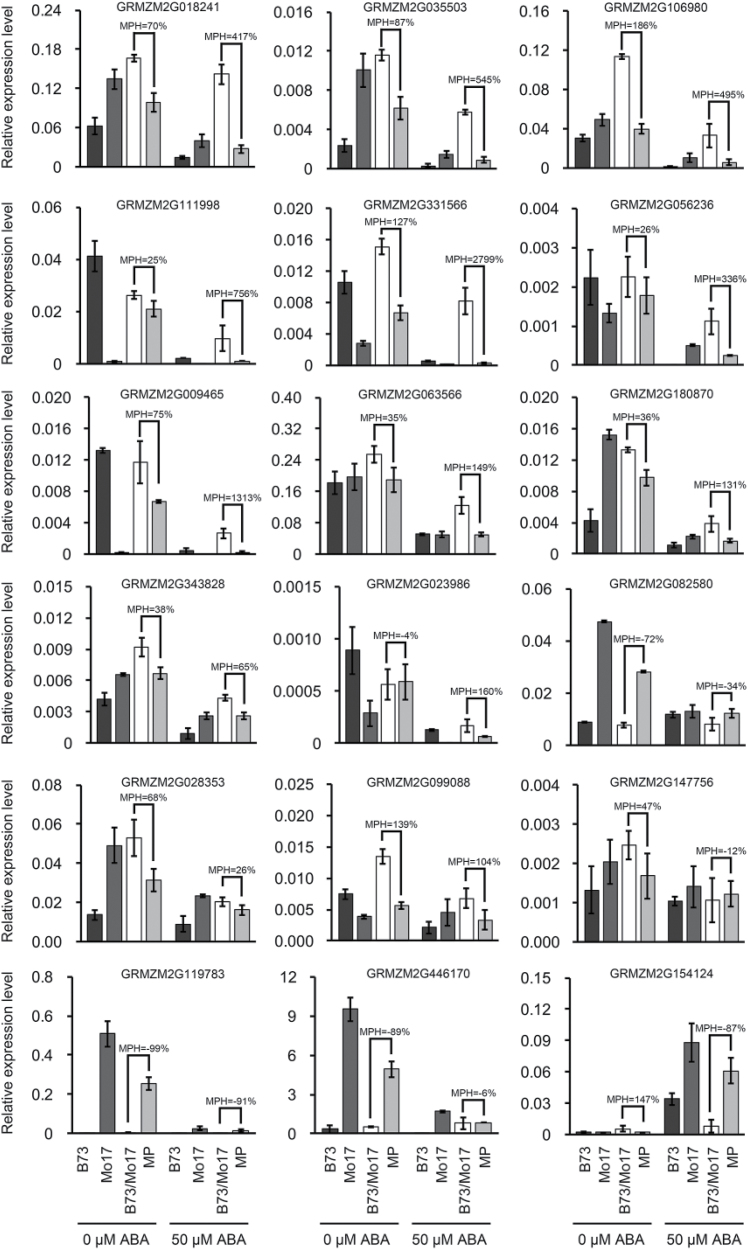
**Effect of ABA on the relative expression levels of non-additive genes related to the cell wall.** Samples consist of embryos of the hybrid B73/Mo17 and its parental inbred lines at 16 HAI with or without ABA treatment. *ZmActin1* was used as an internal control. MP and MPH represent the mid-parent value and mid-parent heterosis, respectively. The MPH was calculated using the following formula: MPH = (F_1_ − MP)/MP in %, where F_1_ is the average value of the hybrid, and the MP is the average value of the two parents.

As an important cellular process for plant growth, cell expansion is a net result of internal turgor pressure and irreversible cell wall extension. This process requires the cell wall to be irreversibly stretched through a wall-loosening process followed by deposition of new wall materials (Cosgrove, 2005). Interestingly, a comparative transcriptome profiling analysis showed that 50 non-additively expressed genes in the seed embryo encoded proteins related to cell wall metabolism (Supplementary Table S2). These data provide further evidence that changes in cell wall loosening and remodelling in relation to cell expansion in the embryo axis are a determinant feature in seed germination heterosis. Remarkably, of 18 examined genes, 13 were confirmed to have above high inbred parent or high inbred parent expression patterns at 16 HAI without ABA treatment. A further 12 of these genes exhibited higher MPH in terms of relative expression level after ABA treatment than that expressed before ABA application (Fig. 8). Collectively, our findings suggest that ABA-mediated seed germination heterosis results from the altered expression of genes involved in cell wall loosening and expansion.

**Fig. 9.**
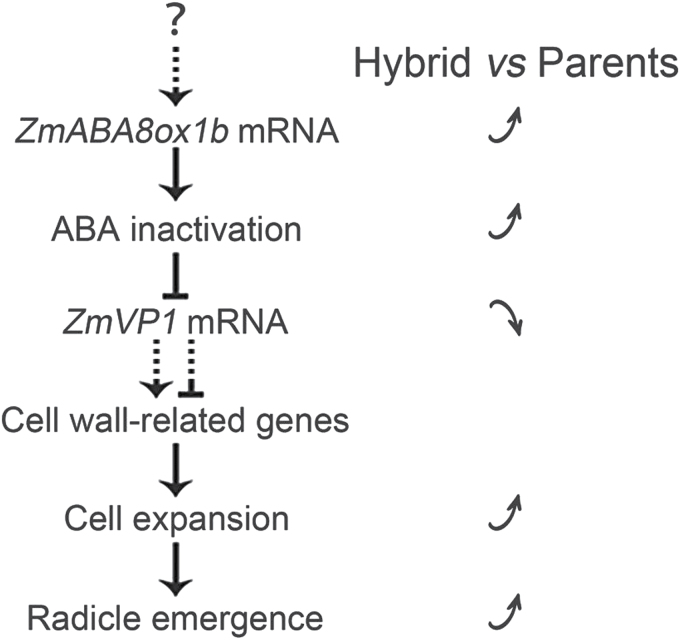
**A proposed model of seed germination heterosis regulated by ABA inactivation and the ABA signalling pathway.** Up arrow and down arrow indicate activation and repression in the hybrid, respectively.

In conclusion, we propose a simple model for ABA-mediated seed germination heterosis (Fig. 9). Briefly, rapid ABA inactivation occurs in hybrids during seed germination due to the up-regulation of *ZmABA8ox1b*, which leads to the down-regulation of *ZmVP1*, a key transcription factor in the ABA signalling pathway. The expression levels of genes involved in cell wall loosening and expansion are then altered, which accelerates cell expansion in hybrids and contributes to the observed seed germination heterosis.

## Supplementary data

Supplementary data are available at *JXB* online.

Figure S1. Relative expression levels of *ZmABA8oxs* in hybrid B73/Mo17 during seed germination.

Figure S2. Overexpression of *ZmABA8ox1b* in *Arabidopsis*.

Figure S3. Microscopic observation of a longitudinal section (A) and cross section (B) of the embryo radicle of hybrid B73/Mo17 at 16 HAI.

Figure S4. Correlation of RNA-seq data between replicates.

Figure S5. Twenty genes were selected to examine the accuracy of RNA-seq using qRT-PCR.

Figure S6. Gene expression patterns of four cell wall-related genes between hybrid B73/Mo17 and its parental inbred lines during seed germination.

Table S1. Gene-specific primer pairs used in this study.

Table S2. Annotation of non-additively expressed genes related to the cell wall.

Supplementary Data
