# Supplementary material for: Up-regulating the abscisic acid inactivation gene ZmABA8ox1b contributes to seed germination heterosis by promoting cell expansion
Source: J Exp Bot. 2016 Mar 31;67(9):2889–900. doi: 10.1093/jxb/erw131 (PMC4861030; doi:10.1093/jxb/erw131)
Supplement: Supplementary Data [file supp_67_9_2889__index.html]

Up-regulating the abscisic acid inactivation gene ZmABA8ox1b contributes to seed germination heterosis by promoting cell expansion — Supplementary Data 

# Up-regulating the abscisic acid inactivation gene *ZmABA8ox1b* contributes to seed germination heterosis by promoting cell expansion

## Supplementary Data

Data files

- Supplementary\_Figures\_S1\_S6\_Table\_S1.pdf - Supplementary Data
- Supplementary\_Table\_S2.xlsx - Supplementary Data
