# Supplementary material for: Up-regulating the abscisic acid inactivation gene ZmABA8ox1b contributes to seed germination heterosis by promoting cell expansion
Source: J Exp Bot. 2016 Mar 31;67(9):2889–900. doi: 10.1093/jxb/erw131 (PMC4861030; doi:10.1093/jxb/erw131)
Supplement: Supplementary Data [file supp_erw131_Supplementary_Figures_S1_S6_Table_S1.pdf]

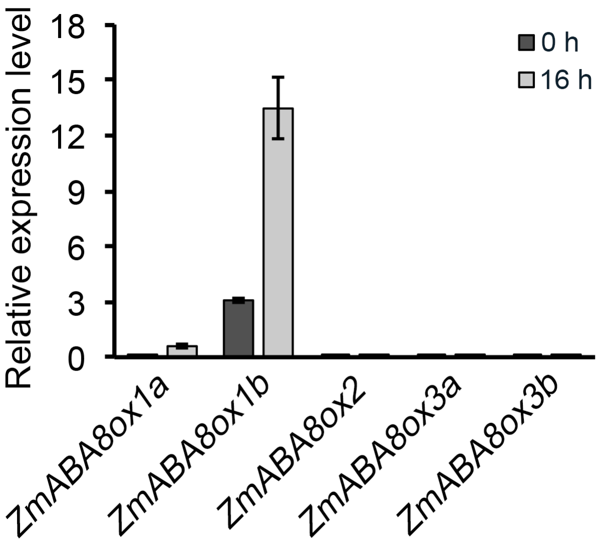

Figure S1. Relative expression levels of *ZmABA8oxs* in hybrid B73/Mo17 during seed germination. *ZmActin1* was used as an internal control.

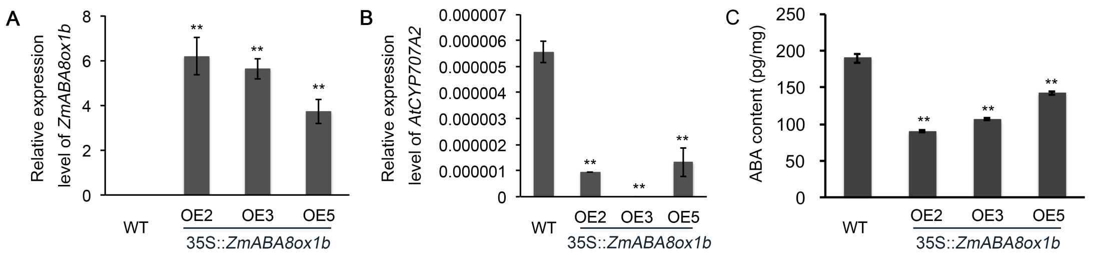

Figure S2. Overexpression of *ZmABA8ox1b* in *Arabidopsis*. (A-B) Relative expression levels of *ZmABA8ox1b* (A) and *AtCYP707A2* (B) in the rosette leaves of wild type (WT) and three 35S::*ZmABA8ox1b* transgenic lines (OE2, OE3 and OE5). *AtActin2* was used as an internal control. (C) ABA content in dry seeds of wild type (WT) and three 35S::*ZmABA8ox1b* transgenic lines (OE2, OE3 and OE5). Data were statistically analysed with a *t*-test: $^{**}P < 0.01$.

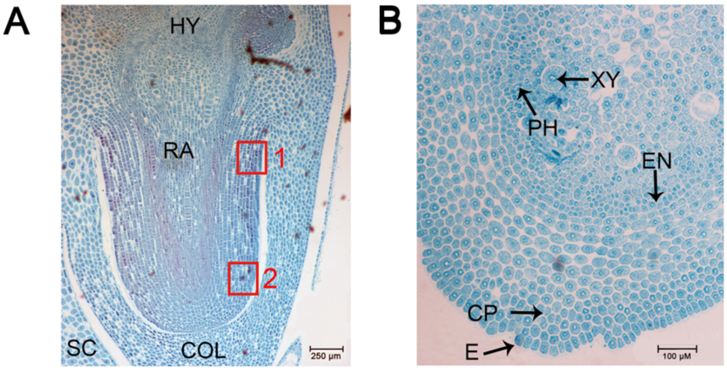

Figure S3. Microscopic observation of a longitudinal section (A) and cross section (B) of the embryo radicle of hybrid B73/Mo17 at 16 HAI. HY, hypocotyl. RA, radicle. SC, scutellum. COL, coleorhiza. XY, xylem. PH, phloem. EN, endodermis. E, epidermis. CP, cortical parenchyma. Labels 1 and 2 represent the upper and apical region of the radicle, respectively.

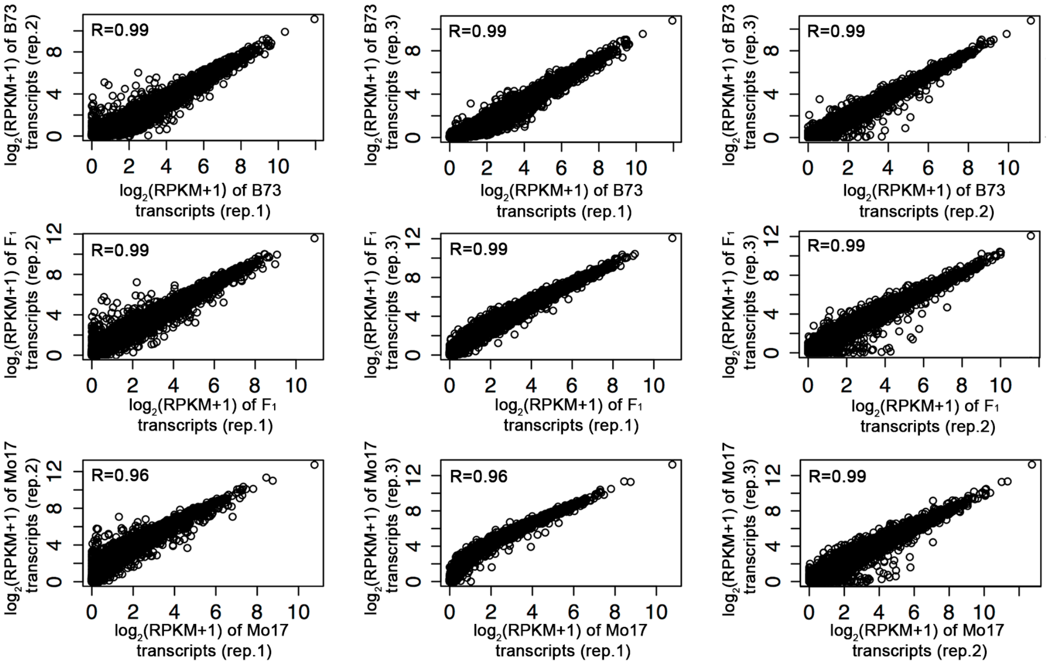

Figure S4. Correlation of RNA-seq data between replicates. F_1 represents the hybrid B73/Mo17. RPKM stands for reads per kilobase of transcript per million mapped reads.

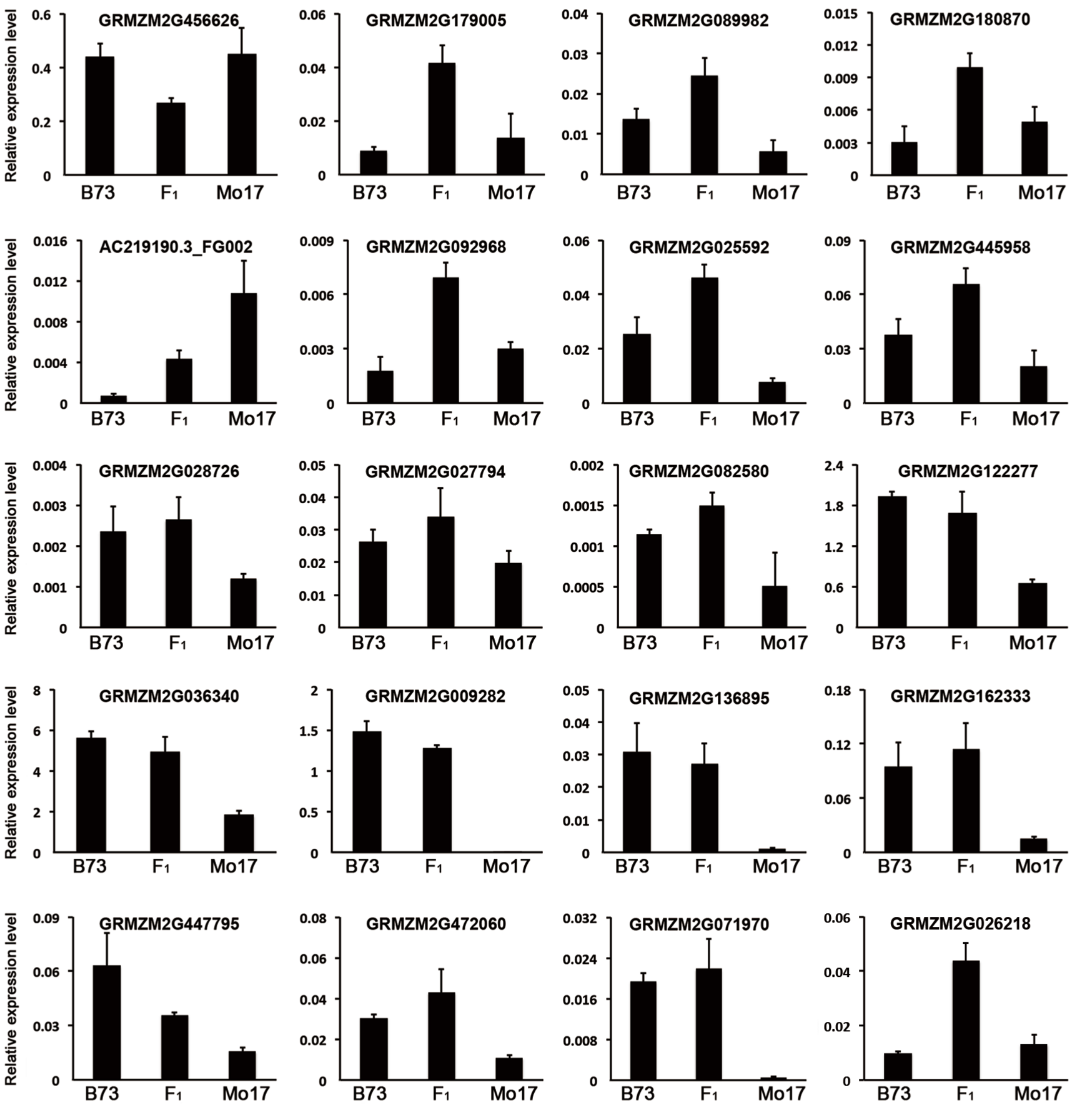

Figure S5. Twenty genes were selected to examine the accuracy of RNA-seq using qRT-PCR. F_1 represents the hybrid B73/Mo17. *ZmActin1* was used as an internal control.

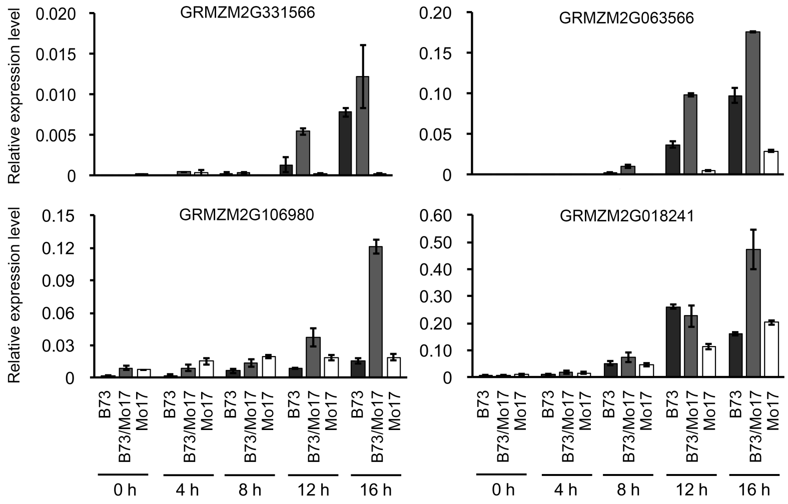

Figure S6. Gene expression patterns of four cell wall-related genes between hybrid B73/Mo17 and its parental inbred lines during seed germination. *ZmActin1* was used as an internal control.

Table S1. Gene-specific primer pairs used in this study.

Name	Sequence	Description
Gateway-1b-1F	5´-AAAGCAGGCTTCCCACCGCAACAAGCAGAGT-3´	Vector construction
Gateway-1b-1R	5´-GAAAGCTGGGTCCGGTTTTTCGCGTTCCTGG-3´	
Gateway-1b-2F	5´-GGGGACAAGTTTGTACAAAAAAGCAGGCTTC-3´	Vector construction
Gateway-1b-2R	5´-GGGGACCACTTTGTACAAGAAAGCTGGGTC-3´	
AtCYP707A2-F	5´-ACCCCTCAATACTTCAAGCAAAAG-3´	qRT-PCR
AtCYP707A2-R	5´-TGAAGACGATGAAGATGAGATTTGC-3´	
ZmABA8ox1a-F	5´-ATGCTCGTGCTCTTCCACCACCT-3´	qRT-PCR
ZmABA8ox1a-R	5´-TATACCGCCATACCATATCCATCCGCC-3´	
ZmABA8ox1b-F	5´-ATGCTCGTGCTCTTCCACCACCT-3´	qRT-PCR
ZmABA8ox1b-R	5´-GGAAGCGGTTTTTCGCGTTCCTGG-3´	
ZmABA8ox2-F	5´-AGCCTACGAGGAGAACGATG-3´	qRT-PCR
ZmABA8ox2-R	5´-TCAGGAACCCTTGGTAGTGC-3´	
ZmABA8ox3a-F	5´-ACAGAAAGGGGCGTGAGACCGA-3´	qRT-PCR
ZmABA8ox3a-R	5´-AGGCGAGCAAAGAAGAATTTCAA-3´	
ZmABA8ox3b-F	5´-ACAGAAATGGCCGCCATGAGACCGA-3´	qRT-PCR
ZmABA8ox3b-R	5´-ATTTCTTCTCCCCCTCAAGGTAAT-3´	
ZmVP1-F	5´-AGAAGGTGCTGAAGCAGAGC-3´	qRT-PCR
ZmVP1-R	5´-CTGTACCGCATGTTCCACAC-3´	
ZmActin1-F	5´- CGATTGAGCATGGCATTGTCA-3´	qRT-PCR
ZmActin1-R	5´- CCCACTAGCGTACAACGAA-3´	
AtActin2-F	5´- TGCAGACCGTATGAGCAAAG-3´	qRT-PCR
AtActin2-R	5´- CCGTCATGGAAACGATGTCT-3´	
GRMZM2G456626-F	5´- GGGACATGACCCAAACGGAA-3´	qRT-PCR
GRMZM2G456626-R	5´- CAGCGTTTGCCATTCATCCA-3´	
GRMZM2G179005-F	5´- CGCTTCTTGTCCTGCAATCG-3´	qRT-PCR
GRMZM2G179005-R	5´- ACAACGCTCTCTTGGATCAGT-3´	
GRMZM2G089982-F	5´- TCGTGATGTTGGTGGCGTAT-3´	qRT-PCR
GRMZM2G089982-R	5´- AGCAGATCTATGGCAACCGA-3´	
GRMZM2G180087-F	5´- GGGTTCGTGTCAAGGACCAA-3´	qRT-PCR
GRMZM2G180087-R	5´- AATGCCTGCACTCACTCACA-3´	
AC219190.3_FG002-F	5´- AGAACTGGCAGTGCGACG-3´	qRT-PCR
AC219190.3_FG002-R	5´- GAACTGCTTGCCCTCGAACG-3´	
GRMZM2G092968-F	5´- ATATGAGCCCCAAAGGAGCTG-3´	qRT-PCR
GRMZM2G092968-R	5´- CATGAGCTCAGGGTGACGAG-3´	
GRMZM2G025592-L	5´- AGGGGAGGTAGTTGAGCAGT-3´	qRT-PCR
GRMZM2G025592-R	5´- CCCTGCGAATCAGCACAATG-3´	
GRMZM2G445958-F	5´- AGGCTTAGCAGCACATTCAGA -3´	qRT-PCR
GRMZM2G445958-R	5´- CCTGTTAGTGGTTCCGGTGG -3´	

Name	Sequence	Description
GRMZM2G028726-F	5´- CGGTGGATGCTTGTGCTAGA-3´	qRT-PCR
GRMZM2G028726-R	5´- GCGACGGGCGTCAAAGTTA-3´	
GRMZM2G027794-F	5´- GGGATCCATGTGATTTGGGC-3´	qRT-PCR
GRMZM2G027794-R	5´- TCGAAGTTTACTGCTGGTGC-3´	
GRMZM2G082580-F	5´- AGGTACATTTCGTTGTGCCGC-3´	qRT-PCR
GRMZM2G082580-R	5´- GCTCGCCTCCATCTCTGTCC-3´	
GRMZM2G122277-F	5´- GATGATATGGTGAGCCGCGA-3´	qRT-PCR
GRMZM2G122277-R	5´- ATGTGTGTGGGTTGGTGTGT-3´	
GRMZM2G036340-F	5´- TAGCTGCTCTGCTCTGCTCT-3´	qRT-PCR
GRMZM2G036340-R	5´- CGTCGTCGATCGATCTTAAT-3´	
GRMZM2G009282-F	5´- CAGTTCACAGTTCACGCTGC-3´	qRT-PCR
GRMZM2G009282-R	5´- GAGGATGGCCGAACGAGAAA-3´	
GRMZM2G136895-F	5´- TGCTGGTGGTGACCAAGAAT-3´	qRT-PCR
GRMZM2G136895-R	5´- GGACGACGCGGACATGGTAT-3´	
GRMZM2G162333-F	5´- CTCCGTCGTCTCAGCGTATG-3´	qRT-PCR
GRMZM2G162333-R	5´- AGCGATGGTGAGGTTGCTTT-3´	
GRMZM2G447795-F	5´- CCAGCAGAAAACATAGAACTTGC-3´	qRT-PCR
GRMZM2G447795-R	5´- ACACCATGAATTGCGTCCGA-3´	
GRMZM2G472060-F	5´- GGTCCCAAGACCTGGAATGG-3´	qRT-PCR
GRMZM2G472060-R	5´- GGATTAGATGGGCCAGAGCC-3´	
GRMZM2G071970-F	5´- CGCGGCAGGAAGGAAAGC-3´	qRT-PCR
GRMZM2G071970-R	5´- CGGCGAGGAGTGGAACTTAAA-3´	
GRMZM2G026218-F	5´- ATCCTTCCTCCCCCTGTCTG -3´	qRT-PCR
GRMZM2G026218-R	5´- CGGACGACCATACAACTAGCA -3´	
GRMZM2G018241-F	5'-ATCCAGTTGCATTACCGTTC-3'	qRT-PCR
GRMZM2G018241-R	5'-CCATACTCCGAAACCAAACA-3'	
GRMZM2G035503-F	5'-ACACGTACAACCCTCCGTTC-3'	qRT-PCR
GRMZM2G035503-R	5'-CCATTGACGCGGTTGTAGGA-3'	
GRMZM2G106980-F	5´- ACAAGATACTGGCGCAGAGG-3´	qRT-PCR
GRMZM2G106980-R	5´- ACCAGAAAGAGTGTGGCGAG-3´	
GRMZM2G111998-F	5´- TCATGCTGGGAGACTACCGA-3´	qRT-PCR
GRMZM2G111998-R	5´- ACGTACGTGGCATTCACCAT-3´	
GRMZM2G331566-F	5´- GGTGACAACGTCAAGTTCGG-3´	qRT-PCR
GRMZM2G331566-R	5´- GACTTCATGAGGCCGCCAAA-3´	
GRMZM2G056236-F	5´- TGACTACTGAACCAGCACCAG-3´	qRT-PCR
GRMZM2G056236-R	5´- ACTTGGTGGGCCAGATTACC-3´	
GRMZM2G009465-F	5´- TCGTCAAAGGGCAACTTCGT-3´	qRT-PCR
GRMZM2G009465-R	5´- TCTTGATCGACCCACACAGG-3´	

Name	Sequence	Description
GRMZM2G063566-F	5´-CTCGTGCTTCTACCTCTCTTCC-3´	qRT-PCR
GRMZM2G063566-R	5´-CAGAGGTCGAACTGGTGCTC-3´	
GRMZM2G180870-F	5´-TGCGCTCCGTTTCAGAGATT-3´	qRT-PCR
GRMZM2G180870-R	5´-AGCTTCAAGTACACCCACCG-3´	
GRMZM2G343828-F	5´-TAGACATCACGTTCGCCCAG-3´	qRT-PCR
GRMZM2G343828-R	5´-CCCACTGGGGTTCTTCTCAC-3´	
GRMZM2G023986-F	5´-TGTTCGAGTGCTTCAGGCAA-3´	qRT-PCR
GRMZM2G023986-R	5´-CCAGCTTGATGGACAGGAGG-3´	
GRMZM2G028353-F	5´- GCCGTGGAGGTGACCCTAAT-3´	qRT-PCR
GRMZM2G028353-R	5´- GGATTCTCTTTCCCCCACCG-3´	
GRMZM2G099088-F	5´- AGCAGATTTGGTGGAGACGG-3´	qRT-PCR
GRMZM2G099088-R	5´- CCTTGTACCCCTTCCGGTTC-3´	
GRMZM2G147756-F	5´- TCAGTTAATGGACACTTCTCTCTCT-3´	qRT-PCR
GRMZM2G147756-R	5´- GTGTGTGTGAATGCGAGCTG-3´	
GRMZM2G119783-F	5´- TAGTATAAAGCCGCGCCACC-3´	qRT-PCR
GRMZM2G119783-R	5´- AGCTTTCTGGGAATCGGTCG-3´	
GRMZM2G446170-F	5´- CGGCCTACTACTCGGCTTC-3´	qRT-PCR
GRMZM2G446170-R	5´- TGTCGGGAAGAGCAGACTTC-3´	
GRMZM2G154124-F	5´- TTCATCGTTCTCCGTGGCTC-3´	qRT-PCR
GRMZM2G154124-R	5´- CGTCTCTCCCAAGCCGAAAT-3´	
